# Network Pharmacology and Bioinformatics Analyses Identify Intersection Genes of Vitamin D3 and COVID-19 as Potential Therapeutic Targets

**DOI:** 10.3389/fphar.2022.874637

**Published:** 2022-04-28

**Authors:** Shanglin Wang, Huayu Gao, Xiaoru Wang, Xiaoli Ma, Lulu Zhang, Yuanxin Xing, Yanfei Jia, Yunshan Wang

**Affiliations:** ^1^ Research Center of Basic Medicine, Jinan Central Hospital, Shandong University, Jinan, China; ^2^ Research Center of Basic Medicine, Jinan Central Hospital, Shandong First Medical University, Jinan, China; ^3^ Department of Pediatric Surgery, The First Affiliated Hospital of Shandong First Medical University, Jinan, China; ^4^ Department of Traditional Chinese Medicine, Jinan Central Hospital, Shandong First Medical University, Jinan, China

**Keywords:** gastric cancer, COVID-19, vitamin D3, calcitriol, network pharmacology, bioinformatics analyses, molecular docking

## Abstract

**Purpose:** The persistent pandemic of coronavirus disease 2019 (COVID-19), the discovery of gastrointestinal transmission routes and the possible susceptibility of cancer patients to COVID-19 have forced us to search for effective pathways against stomach adenocarcinoma (STAD)/COVID-19. Vitamin D3 (VD3) is a steroid hormone with antiviral, anti-inflammatory and immunomodulatory properties. This study aimed to evaluate the possible functional role and potential mechanisms of action of VD3 as an anti-COVID-19 and anti- STAD.

**Methods:** Clinicopathological analysis, enrichment analysis and protein interaction analysis using bioinformatics and network pharmacology methods. Validate the binding activity of VD3 to core pharmacological targets and viral crystal structures using molecular docking.

**Results:** We revealed the clinical characteristics of STAD/COVID-19 patients. We also demonstrated that VD3 may be anti- STAD/COVID-19 through antiviral, anti-inflammatory, and immunomodulatory pathways. Molecular docking results showed that VD3 binds well to the relevant targets of COVID-19, including the spike RBD/ACE2 complex and main protease (Mpro, also known as 3CLpro). We also identified five core pharmacological targets of VD3 in anti-STAD/COVID-19 and validated the binding activity of VD3 to PAI1 by molecular docking.

**Conclusion:** This study reveals for the first time that VD3 may act on disease target gene *SERPINE1* through inflammatory and viral related signaling pathways and biological functions for the therapy of STAD/COVID-19. This may provide a new idea for the use of VD3 in the treatment of STAD/COVID-19.

## Introduction

The coronavirus disease 2019 (COVID-19), currently caused by the infectious severe acute respiratory syndrome coronavirus type 2 (SARS-CoV-2), continues to place unprecedented strain on healthcare systems around the world. As of February 7, 2022, the number of patients has reached 396,252,002, with at least 5.7 million deaths. Unfortunately there is no specific drug for this deadly disease (Tang et al., 2020). Therefore, there is an urgent need for screening and validation of potential anti-COVID-19 targets or drugs. The clinical syndromes in COVID-19 patients usually present as respiratory symptoms, but there is increasing evidence that COVID-19 patients are also accompanied by gastrointestinal (GI) symptoms. And abdominal pain, diarrhea and ulcers are more frequently seen in patients in the intensive care unit than in those in the general care unit (Lin et al., 2020; Pan et al., 2020). It has been shown that SARS-CoV-2 can also be detected in adult stool samples and air samples from patients toilet areas (Liu et al., 2020; Zhang et al., 2020). And that adult gastric-like organs are more susceptible to infection after differentiation (Giobbe et al., 2021). This suggests that the stomach may have an active role in fecal-oral SARS-CoV-2 transmission. Furthermore, it has been shown that cancer patients are more likely to be infected with COVID-19 and have a more severe course of infection than non-cancer patients, due to a state of systemic immunosuppression caused by malignancy and anticancer therapy. As a result, these patients may be at increased risk of COVID-19 infection and have a poorer prognosis (Liang et al., 2020). Gastric cancer is one of the most common cancers and the third leading cause of cancer-related deaths worldwide (Arnold et al., 2020). Among them, the incidence of stomach adenocarcinoma (STAD) accounts for 95% of gastric malignancies. Therefore, effective treatment is urgently needed for COVID-19 patients, especially for STAD patients infected with a novel coronavirus (SARS-COV-2), which may be a new idea to improve patient survival and cut off the fecal-oral transmission route.

Vitamin D3 (VD3) is often used to improve overall health and is a precursor to the potent steroid hormone calcitriol 1,25 dihydroxy VD3 [1,25 (OH) 2D3]. And there is growing evidence that VD3 levels correlate with the risk of a variety of cancers (Feldman et al., 2014), including colon (Pereira et al., 2012), prostate (Mondul et al., 2016; Ferrer-Mayorga et al., 2017) and breast (Swami et al., 2000) cancers. Studies have also shown that VD3 deficiency is associated with a poor prognosis in gastric cancer (Bao et al., 2014). In particular, its deficiency is associated with increased susceptibility to infectious diseases (Martineau et al., 2019). Furthermore, treatment with calcitriol resulted in hepatitis C virus (HCV) suppression (Gal-Tanamy et al., 2011). This suggests that VD3 has a natural and direct antiviral effect. Interestingly, we also found evidence that VD3 against helicobacter pylori (*H. pylori*) and calcitriol showed beneficial anti-inflammatory effects in cancer (Krishnan and Feldman, 2011; Hu et al., 2019; Zhou et al., 2020). Particularly, VD3 was found to increase the expression of angiotensin-converting enzyme (ACE2), the functional receptor of SARS-CoV-2. Furthermore, studies suggested the possible protective role of VD3 on lung by inducing ACE2/Ang-(1-7)/MasR axis activity and inhibiting renin and ACE/Ang II/AT1R axis (Xu et al., 2017; Ganji et al., 2020). Although research findings have shown a correlation between low vitamin D levels and risk of death and COVID-19 infection (Maghbooli et al., 2020; Merzon et al., 2020), there is no consensus on the role of VD3 in COVID-19 outcomes (Cannata-Andía et al., 2022). Therefore, the pharmacological targets and mechanisms of VD3 or calcitriol against STAD/COVID-19 remain to be studied in detail.

Based on public databases and publicly available data, network pharmacology plays an important role in discovering biologically active ingredients, predicting drug targets, and analyzing drug mechanisms of action (Hopkins, 2008). In this study, bioinformatics and network pharmacology strategies, combined with the molecular docking approach, were used to explore the active components and potential targets of VD3 against STAD/COVID-19 for network visualization. We also created a flow chart to demonstrate the mechanism of VD3 resistance to STAD/COVID-19.

## Materials and Methods

### Identification of STAD/COVID-19–Associated Genes

Transcriptome profiles of STAD patients were downloaded from The Cancer Genome Atlas (TCGA) database in November 2021. The differential genes in STAD were screened and obtained using the ‘limma’ package of R-language Bioconductor. Furthermore, genes related to COVID-19 were selected from the OMIM database, Genecard database, KEGG and NCBI databases. Finally, these genes were compared to obtain the overlapping genes in STAD and COVID-19 (Li et al., 2020; Li et al., 2021a).

### Collection of VD3 Related Genes

VD3-related genes were collected from accessible online tools, such as Traditional Chinese Medicine Systems Pharmacology Database and Analysis Platform (TCMSP), Swiss Target Prediction, TargetNet and Drugbank. Protein-gene name conversion was then performed using the protein database UniProt. Finally, a list of related genes was obtained by removing duplicates of these genes.

### Clinicopathological Analysis of STAD and COVID-19–Associated Genes

The correlation of STAD/COVID-19-related genes with survival in STAD/COVID-19 patients was analyzed in the ‘survival package’ of the R package. Prognostic analysis of STAD/COVID-19 patients was performed using univariate and multivariate Cox proportional hazards regression. In addition, we analyzed the different characteristics of patients with STAD/COVID-19 (Fisher and Lin, 1999).

### Enrichment Analyses and Network Visualization

Using R-language packages, including ‘ClusterProfiler', ‘org.Hs.eg.Db’, ‘pathview’, and ‘GOplot’, etc. for enrichment analysis and visualization of intersecting genes by Gene Ontology (GO) and Kyoto Encyclopedia of Genes and Genomes (KEGG). GO enrichment analysis annotated the mechanism of action of target genes in three parts: biological process (BP), cellular composition (CC) and molecular function (MF), and analyzed the top ten entries of each part according to their *p* values. KEGG enrichment analysis was performed to annotate the signaling pathways in which the target genes were involved.

### Identifying Core Targets of VD3 Against STAD/COVID-19

Protein-protein interaction network (PPI) and TSV files were obtained by importing the intersecting genes into the STRING database. Next, the top five proteins of the network in terms of attribute ranking were obtained using the MCC calculation method of the cytoHubba plugin in Cytoscape software (Chin et al., 2014).

### Molecular Docking

The protein structures of COVID-19 associated proteins and target proteins were obtained from the PDB database (https://www.rcsb.org/) (Zardecki et al., 2022). The molecular structures of VD3 and Calcitriol were obtained from the PubChem database (https://pubchem.ncbi.nlm.nih.gov/) (Li et al., 2021a). The corresponding proteins and small molecules were processed and docked using Discovery studio-2019 (DS). To ensure the accuracy of the docking prediction results, we used DeepSite to predict the possible docking pockets of the target proteins. DeepSite is freely available at www.playmolecule.org (Jiménez et al., 2017). DeepSite considers various molecular descriptors related to the protein through the 3D-deep convolutional neural networks (DCNNs) validated using an extensive test set based on over 7,000 proteins from the scPDB database (Kellenberger et al., 2006; Jiménez et al., 2017). The cartesian coordinates of the center of the binding pocket found by DeepSite were used to define site in the DS with a radius of 12. And for other parameters, defaults were used. CDOCKER was used for small molecular-protein docking, Top Hit was set to 10, Pose Cluster Radius was set to 0.5. Finally, the interaction and binding modes of the active compounds were visualized and analyzed using PyMoL-2.1.0 and DS.

## Results

### Identification of STAD/COVID-19 Target Genes

**FIGURE 1:**
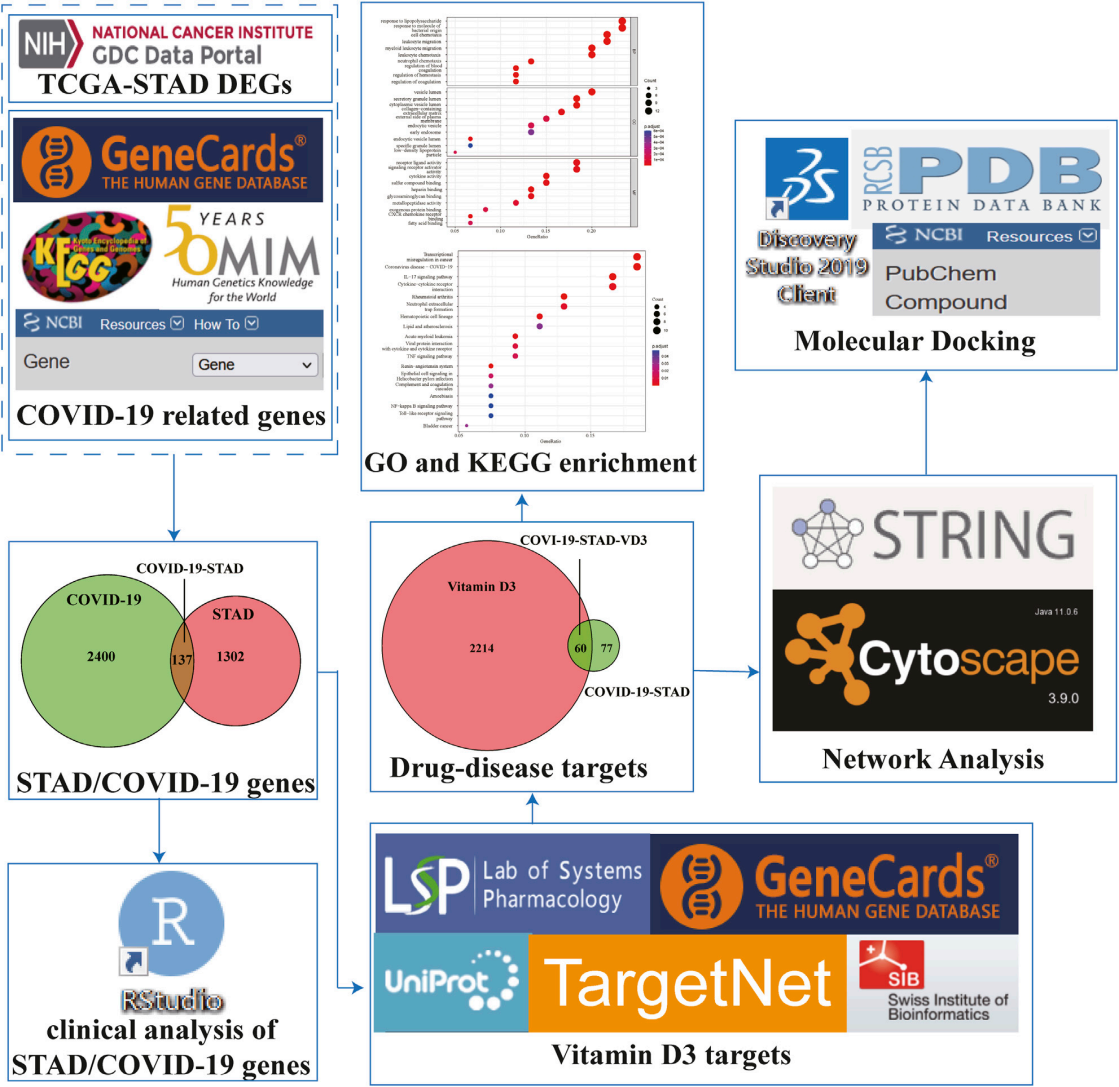
Workflow to investigate the anti-STAD/COVID-19 action and mechanism of vitamin D3.

**FIGURE 2:**
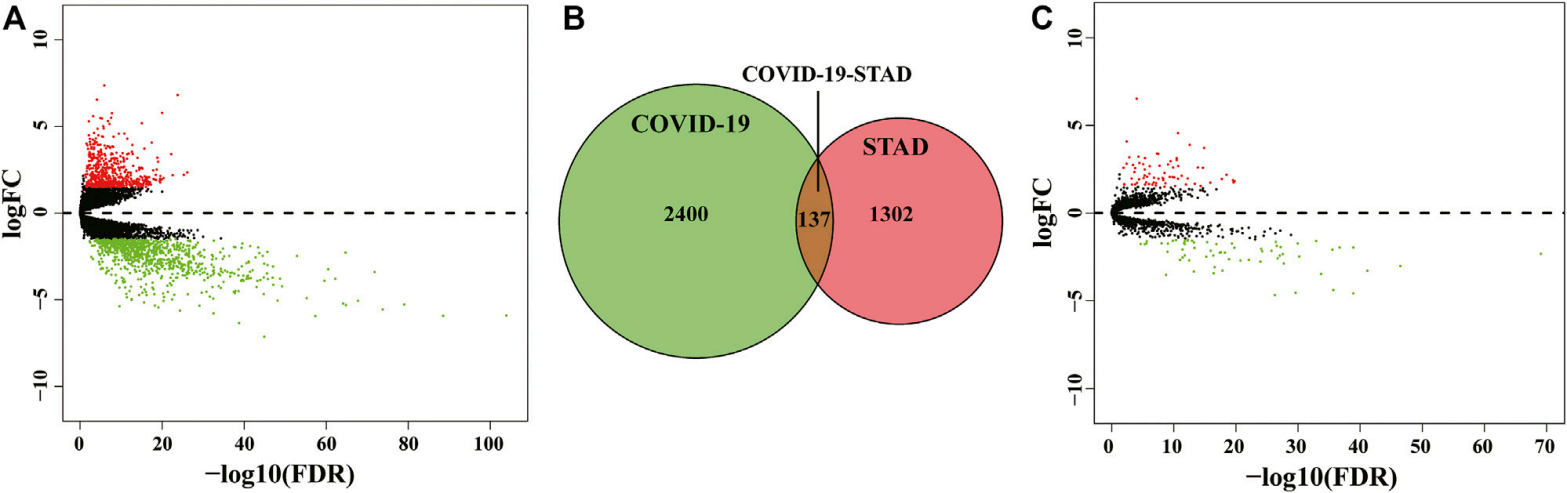
Identification of STAD/COVID-19 target genes. **(A)** Volcano map of differential gene expression associated with STAD. **(B)** Venn diagram depicting intersecting genes in STAD/COVID-19. **(C)** Volcano plot of differential gene expression of STAD and COVID-19 intersecting genes.

The flow of the study was exhibited in Figure 1. From The Cancer Genome Atlas (TCGA) database, we obtained 32 normal samples and 375 tumor samples. When duplicated genes were excluded from all samples, the obtained genes were processed with the egdeR package for normalization and differential expression analysis. An adjusted *p*-value of < 0.05 and a fold change value higher than 1.5 was used to indicate genes that were significantly differentially expressed. We obtained 1,439 differentially expressed genes (DEGs) after analysis and mapped the volcano plot (Figure 2A). After that, we collected 2,537 genes associated with COVID-19 from the Genecard, OMIM, KEGG and NCBI databases by the method of network pharmacology. Comparing these two gene clusters, we identified 137 intersection genes for STAD and COVID-19 (Figure 2B). Of them, 71 were upregulated, and the others were downregulated (Figure 2C).

### Clinicopathological Analysis of STAD/COVID-19-Related Genes

To understand the correlation between STAD/COVID-19-related genes and prognosis, univariate and multifactorial Cox analyses were performed on 137 DEGs. First, univariate Cox analysis identified 15 genes that were significantly (*p* < 0.05) associated (Table 1). However, multivariate Cox analysis identified 4 of these genes, including *KIT*, *HBA1*, *SERPINE1* and *FKBP10* (Table 2). Results in table 2 showed that the 4 genes were bad prognostic factors. In addition, hazard ratios (HR) and 95% confidence intervals (CI) for risk scores from univariate Cox (uni-Cox) regression was 1.21 and 1.136–1.29, respectively, (*p* < 0.001). In comparison, the values of multivariate Cox (multi-Cox) regression were 1.189 and 1.115–1.268, respectively, (*p* < 0.001) (Supplementary Figures S1A,B). In addition, we found two other independent prognostic parameters: age (1.033 and 1.015–1.052; *p* < 0.001) and stage (1.586 and 1.115–1.268; *p* < 0.001) (Supplementary Figure S1B).

**TABLE 1:** Univariate Cox proportional hazards regression analysis.

GENE	HR	HR.95L	HR.95H	*p*-value
*ADAMTS1*	1.020758	1.005256	1.036498	0.008505
*AGT*	1.002566	1.00059	1.004546	0.010895
*APOB*	1.007883	1.000705	1.015113	0.031303
*CSF2*	1.043598	1.000148	1.088935	0.049207
*CXCR2*	1.06722	1.001008	1.137811	0.046503
*CYBRD1*	1.00769	1.00057	1.014861	0.03422
*ERBB2*	1.00069	1.000034	1.001348	0.039318
*F5*	1.012415	1.000812	1.024152	0.035911
*FKBP10*	1.001062	1.00017	1.001955	0.019678
*HBA1*	1.024083	1.008183	1.040234	0.002876
*KIT*	1.051295	1.01247	1.091608	0.009175
*MKI67*	0.983793	0.968023	0.99982	0.0475
*P3H4*	1.002645	1.000167	1.00513	0.036413
*S100A12*	1.005972	1.000591	1.011382	0.02956
*SERPINE1*	1.001841	1.000532	1.003153	0.005846

**TABLE 2:** Multivariate Cox proportional hazards regression analysis.

GENE	coef	HR	HR.95L	HR.95H	*p*-value
*KIT*	0.056912	1.058563	1.017629	1.101143	0.004677
*HBA1*	0.024432	1.024733	1.006387	1.043413	0.008033
*SERPINE1*	0.001765	1.001767	1.000222	1.003314	0.024971
*FKBP10*	0.001049	1.001049	1.000091	1.002009	0.031815

**FIGURE 3:**
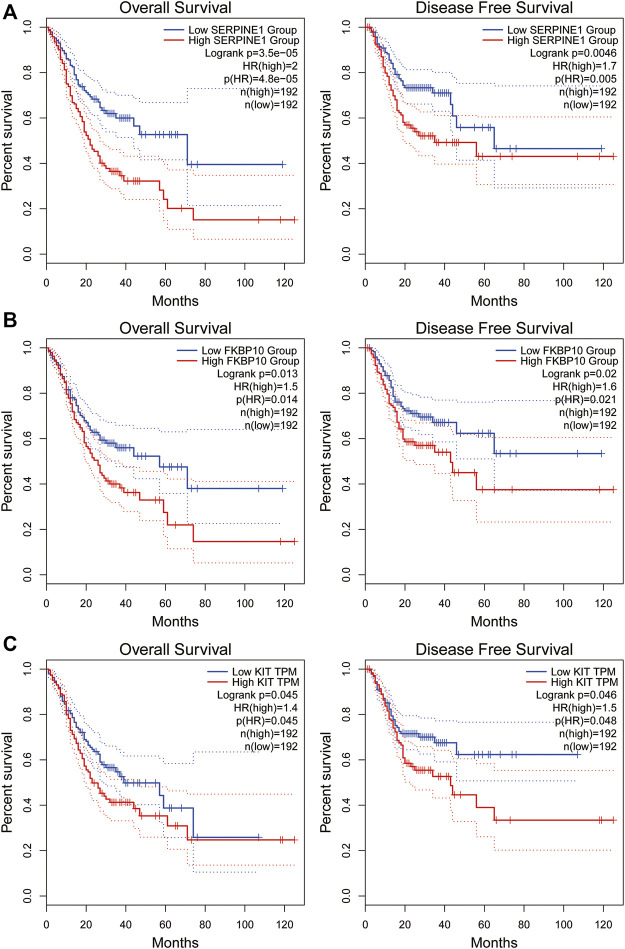
Overall survival and disease-free survival analysis of STAD/COVID-19-related genes in GEPIA datasets. **(A)**: SERPINE1, **(B)**: FKBP10, **(C)**: KIT. *P* < 0.05 was considered statistically significant.

We investigated the correlation between the expression of the four genes obtained in Table 2 and the prognosis of STAD patients through the GEPIA online platform (http://gepia2.cancer-pku.cn/#index). We found that STAD patients with lower *SERPINE1* levels exhibited significantly longer overall survival (OS) and disease-free survival (DFS) than those with higher *SERPINE1* levels (*p* < 0.01). Likewise, STAD patients with lower levels of *FKBP10* or *KIT* showed a better prognosis (*p* < 0.05, Figure 3). The above results indicated that *SERPINE1*, *FKBP10* and *KIT* were closely related to the prognosis of STAD patients.

### Identification of Potential Target Genes of VD3 Against STAD/COVID-19

**FIGURE 4:**
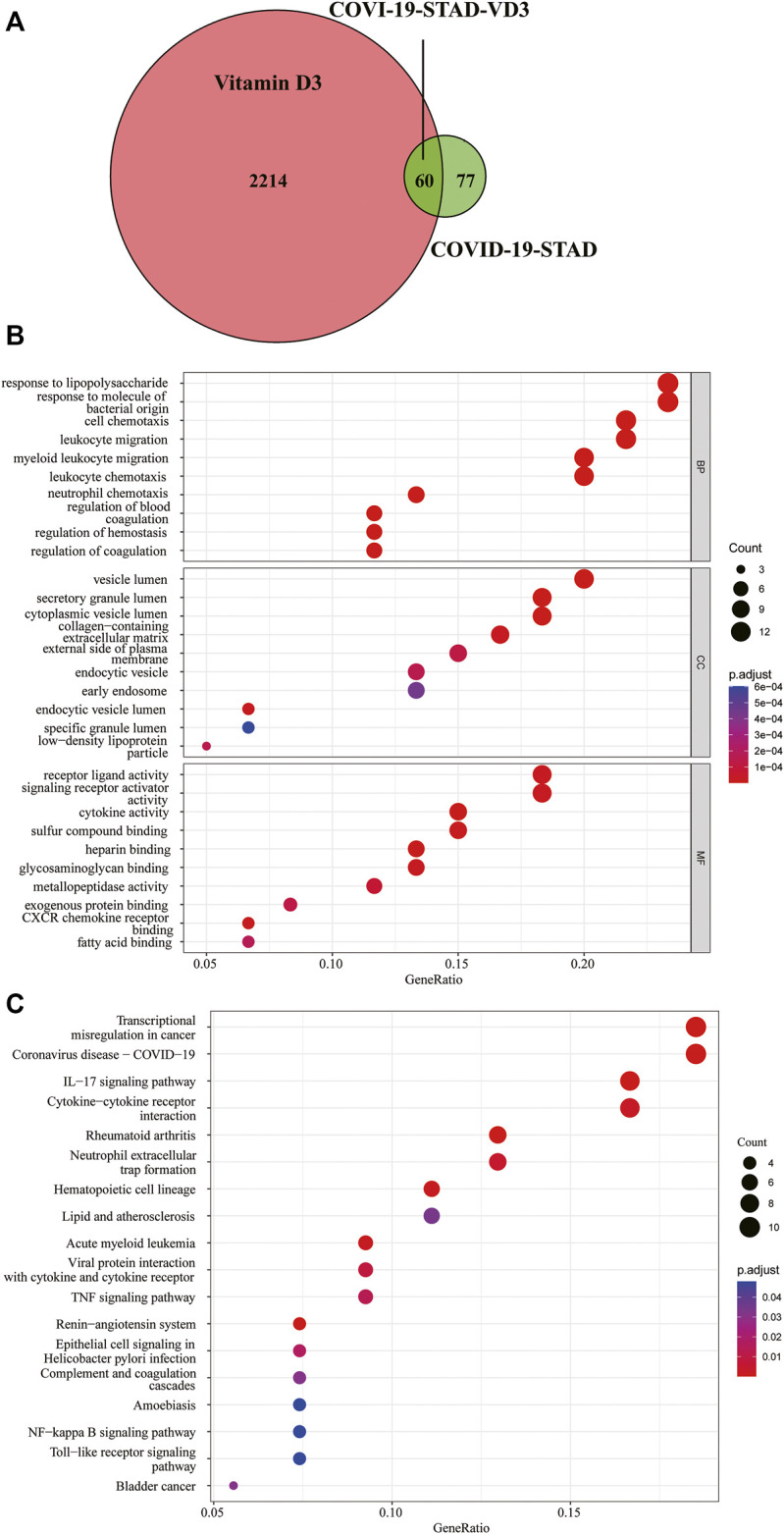
Functional characterization of VD3 against STAD/COVID-19. **(A)** Venn diagram of VD3 and STAD/COVID-19 Intersecting genes. **(B)** GO analysis of intersecting genes of VD3 and STAD/COVID-19. **(C)** KEGG pathway enrichment analysis of intersecting genes of VD3 and STAD/COVID-19.

We downloaded the 3D molecular structure and SMILES [CC(C)CCC​C(C)C1CCC2C1(CCC​C2 = CC = C3CC(CCC3 = C)O)C] of VD3 from PubChem. The PubChem CID of VD3 is 5280795. The chemical formula and molecular weight of VD3 are C_27_H_44_O and 384.6 g/mol, respectively. Then, a portion of the major target genes of VD3 were searched from the Swiss Target Prediction databases and TargetNet web server. Meanwhile, we also collected a portion of VD3-related target genes from GeneCards databases and TCMSP databases. After name conversion of the targets and removing duplicates, 2274 VD3-related target genes were obtained. Identification of 60 VD3 intersecting genes against STAD/COVID-19 after overlap of STAD/COVID-19-related genes with VD3-related target genes (Figure 4A, Supplementary Table S1). By the same method, we identified 52 calcitriol anti- STAD/COVID-19 intersecting genes Supplementary Figure S2A.

For a further, more comprehensive and macroscopic understanding of the biological functions of the intersection genes, we performed GO functional enrichment analysis and KEGG pathway enrichment analysis on these 60 genes. The results showed that VD3 affected 477 biological processes (BP), 32 cellular components (CC), 56 molecular functions (MF) (*p* < 0.05, Supplementary Table S2), involving response to lipopolysaccharide, response to molecule of bacterial origin, cell chemotaxis, leukocyte migration, and other biological processes; vesicle lumen, secretory granule lumen, cytoplasmic vesicle lumen, and other cellular components; receptor ligand activity, signaling receptor activator activity, and other molecular functions (Figure 4B). The 10 biological process terms with the lowest adjusted *p* values in each category are shown in the dot plot. And the above results implied the possible main functions of the 60 intersecting genes.18 signaling pathways associated with all intersection genes were obtained by the KEGG pathway enrichment analysis (*p* < 0.05, Supplementary Table S3), and shown in dot plot according to the gene ratios (Figure 4C). The results showed 60 intersecting genes focused on transcriptional misregulation in cancer, coronavirus disease-COVID-19, IL-17 signaling pathway and cytokine-cytokine receptor interaction pathway. The above results implied the main pathways involved in the 60 intersecting genes. Notably, *SERPINE1* and *FKBP10* genes were found in the hematopoietic cell lineage (hsa04640), acute myeloid leukemia (hsa05221) and complement and coagulation cascades (hsa04610) pathways. Taken together, these results demonstrated that VD3 and calcitriol (Supplementary Figures S2B,C) act on COVID-19 and STAD through multiple pathways, multiple targets, and overall cooperation.

### Identification of Core Targets of VD3 Against STAD and COVID-19

**FIGURE 5:**
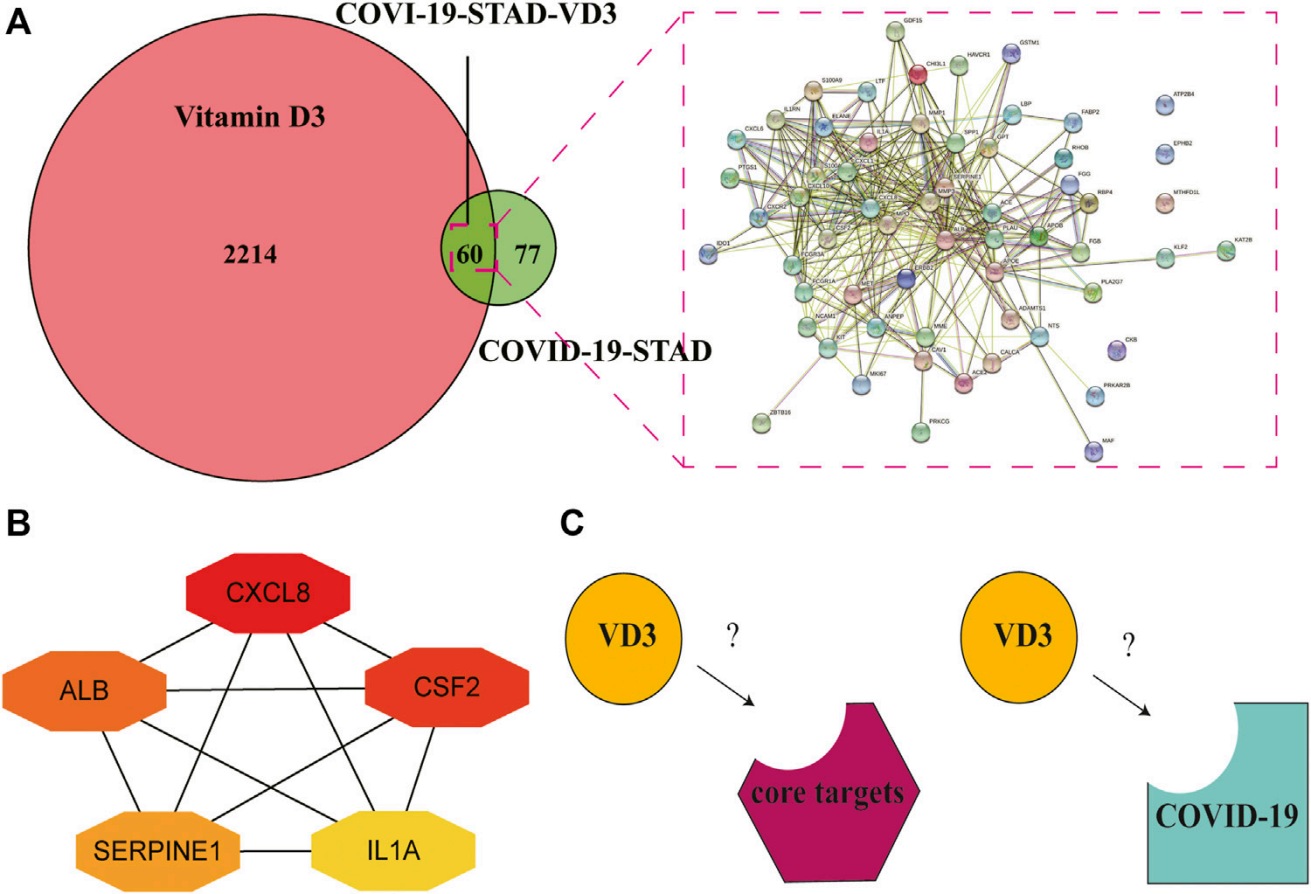
Identification of core targets of VD3 against STAD and COVID-19. **(A)** STRING analysis of VD3 and STAD/COVID-19 intersecting genes. **(B)** Cytoscape analysis representing the gene interaction networks associated with VD3 anti-STAD/COVID-19. The top five hub genes—*CXCL8*, *CSF2*, *ALB*, *SERPINE1* and *IL1A*—are highlighted. **(C)** Diagram of receptor and ligand (VD3 or calcitriol).

Determination of the 60 intersecting target gene-mediated PPI-networks of VD3 against COVID19/STAD using STRING analysis (Figure 5A). Ranked network centrality nodes using the Maximal Clique Centrality (MCC) algorithm of the cytoHubba plugin with 60 intersection genes input to Cytoscape software. Figure 5B showed the connectivity of the top five hub nodes in the network, the darker the node color means the higher the score. The results showed that the top five core target genes were *IL1A*, *CXCL8*, *ALB*, *CSF2* and *SERPINE1*. Furthermore, the hub nodes appeared in 16 pathways in Figure 4C except for neutrophil extracellular trap formation and renin-angiotensin system pathways (Supplementary Table S3).

### Confirmation of VD3 Binding to STAD/COVID-19 Targets

Molecular docking was performed to visualize the patterns of interactions between the VD3 and the identified STAD/COVID-19 target proteins (Figure 5C). We selected the SARS-COV-2 Spike RBD/ACE2 complex (PDB:6LZG) (Wierbowski et al., 2021) and main protease complex crystal structure of COVID-19 (PDB:5R84) (Li et al., 2021a) for molecular docking analysis with VD3. These crystal structures of the COVID-19 were collected from the PDB database (http://www.rcsb.org). In order to predict the VD3-protein binding site, we performed binding pocket simulations of the target structures in this study by referring to the methods applied in the literature for natural product binding mode studies (Singh et al., 2021). In this paper, pockets were predicted using DeepSite and pocket locations were visualized using PyMoL (Supplementary Figure S4). Supplementary Table S4 shows the scores for each prediction pocket.

**FIGURE 6:**
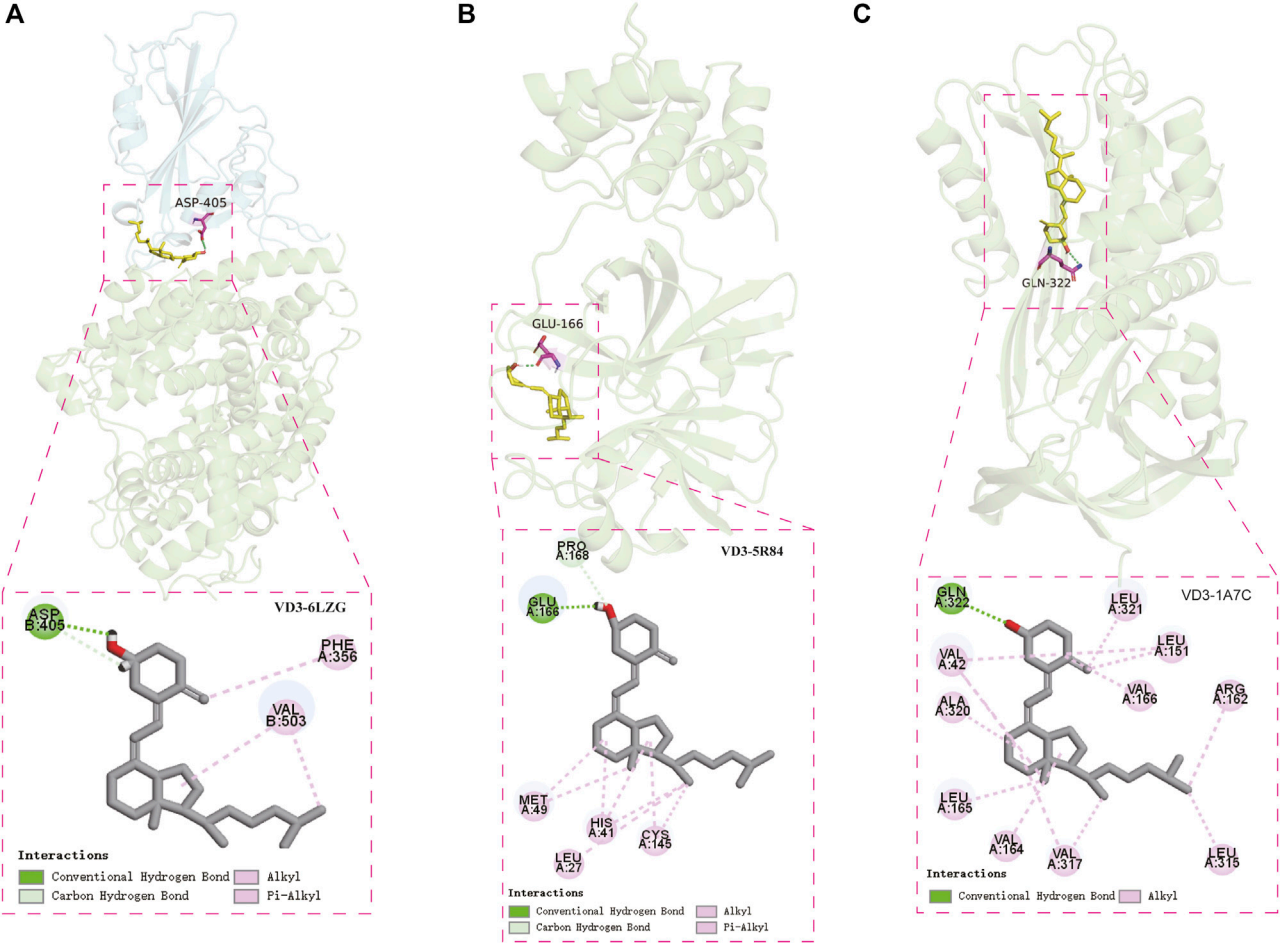
Confirmation of vitamin D3 binding to STAD/COVID-19 targets. **(A)** Molecular docking model of VD3 with SARS-COV-2 Spike RBD/ACE2 complex [PDB: 6LZG, ACE2 (green), SARS-CoV-2 RBD (cyan)] and 2D patterns of bonds in the model. **(B)** Molecular docking model of VD3 with SARS-CoV-2 major protease (PDB: 5R84) and 2D patterns of bonds in the model. **(C)** Molecular docking model of VD3 with PAI1 (PDB: 1A7C) and 2D patterns of bonds in the model.

Two cavity coordinates were detected by DeepSite in SARS-CoV-2 RBD/ACE2 (Supplementary Figure S4A). We believed that interference in protein-protein interactions occurs mainly on the protein surface, and the key amino acid residue (Y41, K353, N501, Y505) reported in the literature that affects the binding of Spike (S) glycoprotein to ACE2 is located near cavity 2 (Supplementary Figure S5) (Lan et al., 2020), so we selected cavity 2 for molecular docking. Our results showed that the hydroxyl group of VD3 forms hydrogen bond with D405 of SARS-CoV-2 RBD/ACE2, and hydrophobic interactions were also found. (Figure 6A). Notably, the binding pocket is located in the protein-protein interaction region of the SARS-COV-2 Spike RBD/ACE2 complex. Based on the docking results, we hypothesized that VD3 may play an inhibitory role in the binding of the Spike glycoprotein of SARS-CoV-2 to the host angiotensin-converting enzyme 2 (ACE2) receptor. We also did the molecular docking of calcitriol with SARS-CoV-2 RBD/ACE2. The results showed that the hydroxyl groups of calcitriol form hydrogen bonds with G504 and Q325 of SARS-CoV-2 RBD/ACE2 (Supplementary Figure S6A). Four cavity coordinates were detected by DeepSite in SARS-CoV-2 Mpro. We selected the highest scoring cavity 2 for molecular docking (Supplementary Figure S4B). The results showed that the hydroxyl group of VD3 forms hydrogen bond with E166 of Mpro, and the hydrophobic interactions were also found. (Figure 6B). Similarly, we did the molecular docking of calcitriol with Mpro. The results showed that the hydroxyl groups of calcitriol could also form hydrogen bonds with Q189, T190, T26 of Mpro (Supplementary Figure S6B). Our results also showed that the amino acid residue VD3 bound to Mpro was consistent with the amino acid residue bound the proto-ligand 2-cyclohexyl-*N*-(pyridin-3-yl) acetamide (GWS) to Mpro, which was E166 (Supplementary Figure S6D).

In addition, we analyzed the possible binding between VD3 and the top 5 STAD/COVID-19 core target proteins (IL1A, IL8, ALBU, CSF2, PAI1) identified in the STRING analysis. Similarly, we also considered the binding of calcitriol. Our result showed that VD3 only bind to PAI1, as does calcitriol. Four cavity coordinates were detected by DeepSite in PAI1. We selected the highest scoring cavity 1 for molecular docking (Supplementary Figure S4C). The hydroxyl group of VD3 was bound to Q322 *via* 1 hydrogen bonding to PAI1 (PDB: 1A7C). And hydrophobic interactions were also found (Figure 6C). The hydroxyl groups of calcitriol were bound to N167, Q322, R163 and L163 of PAI1 by hydrogen bonding (Supplementary Figure S6C). The scores of each docking posture were listed in Table 3.

**TABLE 3:** CDOCKER scores of SARS-CoV-2 and PAI1.

Ligand Name	Target	PBD ID	CDOCKER Interaction Energy (kcal/mol)
Vitamin D3	SARS-CoV-2 RBD/ACE2	6LZG	−27.9718
Calcitriol	SARS-CoV-2 RBD/ACE2	6LZG	−32.8289
Vitamin D3	SARS-CoV-2 Mpro	5R84	−40.8162
Calcitriol	SARS-CoV-2 Mpro	5R84	−50.7525
Vitamin D3	PAI1	1A7C	−54.3729
Calcitriol	PAI1	1A7C	−64.9648

## Discussion

Since the first case of severe acute respiratory syndrome coronavirus 2 (SARS-CoV-2) infection was reported in 2019, infections and mortality rates have continued to rise globally. In addition, the specific therapy for COVID-19 is still inconclusive. The SARS-CoV-2 pandemic has proven to be catastrophic. Therefore, it is urgent to find safe and effective anti-COVID-19 drugs and effective targets. SARS-CoV-2 is commonly thought to be transmitted by the respiratory droplet route, but studies have shown that humans may also be infected with SARS-CoV-2 through the gastrointestinal tract (Lamers et al., 2020; Lin et al., 2020; Pan et al., 2020; Wang et al., 2020; Jiao et al., 2021). Surprisingly, sputum and feces samples from the COVID-19 patients continued to be positive even after their pharyngeal samples changed from positive to negative (Chen et al., 2020). With the trend of the COVID-19 pandemic, hospitals have become a high-risk site for SARS-COV-2 infection, so hospitalized patients have become a high-risk group. Among them, the risk of SARS-COV-2 infection in cancer patients has increased dramatically due to factors such as immunosuppression and immune dysfunction (Gonzalez et al., 2018; Liang et al., 2020). This forced us to pursue a new approach to treating STAD/COVID-19.

VD3 is the active form of vitamin D with the highest rate of biometabolism and is primarily synthesized by the body itself. VD3 is converted by UV irradiation of 7-dehydrocholesterol, which is found in the epidermis and dermis of most advanced animals. And VD3 is subsequently activated to its powerful hormone form, calcitriol (Gil et al., 2018). Based on the beneficial effects of VD3 against gastric cancer (Bao et al., 2014; Li et al., 2017), Helicobacter pylori (Hu et al., 2019), anti-inflammatory actions (Krishnan and Feldman, 2011) and hepatitis C virus (Gal-Tanamy et al., 2011), we hypothesized that VD3 and calcitriol may have effective pharmacological activity in patients with STAD combined with COVID-19. In this work, we used bioinformatics, network pharmacology and molecular docking to elucidate the molecular biological mechanisms of VD3 or calcitriol for the treatment of STAD/COVID-19. This may provide a new idea to improve the survival rate of STAD/COVID-19 patients and to cut off the transmission of SARS-COV-2 through the gastrointestinal tract.

In this study, 137 intersecting genes of COVID-19 and STAD were obtained through the collection and screening of several databases, of which 71 genes were up-regulated and 66 genes were down-regulated. Notably, four important differential genes were identified based on the results of multivariate Cox proportional risk regression analysis, including *KIT*, *HBA1*, *SERPINE1* and *FKBP10*. We also performed survival curve analysis, and the results revealed that patients with low *SERPINE1*, *FKBP10* and *KIT* levels had better DFS and OS than those with high levels (*p* < 0.05). Therefore, the expression of *SERPINE1*, *FKBP10* and *KIT* could be used to predict the prognosis of STAD patients. Next, we combined the 137 DEGs obtained from STAD/COVID-19 samples with VD3-related genes to obtain 60 intersecting genes, and further GO enrichment analysis and KEGG pathway enrichment analysis were performed on these 60 intersecting genes. Similarly, we also considered genes associated with calcitriol. The results suggested that the anti-STAD and anti-COVID19 effects of VD3 were achieved through antiviral effects, anti-inflammatory effects, immunomodulation, and modulation of related signaling pathways. These results suggested that the 60 intersecting genes we obtained may be effective pharmacological targets of VD3 against COVID-19 and STAD. In addition, we found that the anti-COVID-19 and anti-STAD effects of VD3 may be associated with core genes such as *IL1A*, *CXCL8*, *ALB*, *CSF2*, and *SERPINE1*. Next, we speculated that the anti-COVID-19/STAD effects of VD3 might be associated with the core genes *IL1A*, *CXCL8*, *ALB*, *CSF2* and *SERPINE1*, and then did molecular docking to verify the speculation.

The process of SARS-CoV-2 infection could be divided into three steps: attachment and cell entry (S protein and ACE2) (Wrapp et al., 2020; Xu et al., 2020), replication and transcription (Mpro) (Li et al., 2021b), the assembly and release of the mature virus. Therefore, we selected SARS-COV-2 Spike RBD/ACE2 complex (PDB: 6LZG) and SARS-COV-2 Mpro (PDB: 5R84) for molecular docking to simulate the infection process of SARS-COV-2. The results indicated that VD3 had binding activity with SARS-COV-2 RBD/ACE2, Mpro and PAI1. Similarly, we did molecular docking of calcitriol with SARS-COV-2 RBD/ACE2, Mpro and PAI1. These results indicated that either VD3 or calcitriol can effectively bind to specific proteins in the novel coronavirus and has the potential to intervene in the infection process of SARS-CoV-2. Plasminogen activator inhibitor-1 (*PAI1*, aka *SERPINE1*) is a typical member of the Serpin family of proteins (Kellici et al., 2021). *SERPINE1* acts as an inhibitor of tissue plasminogen activator (tPA) and urokinase plasminogen activator (uPA), preventing plasmin formation and inhibiting fibrinolysis and clot dissolution (Wu et al., 1995; Urano et al., 2019). Not surprisingly, *SERPINE1* was identified in the complement and coagulation cascades pathway by KEGG pathway enrichment analysis. A growing number of studies have shown that complement and coagulation cascades direct thrombotic activities in severe COVID-19 patients and also activate neutrophil degranulation (Ibrahim et al., 2004). Neutrophil degranulation and activation of complements of the complement and coagulation pathway can eventually lead to acute respiratory distress syndrome (ARDS), lung injury, thrombosis and other adverse consequences in COVID-19 patients (Jose and Manuel, 2020). In addition, a growing body of evidence has demonstrated that *SERPINE1* expression is associated with poor prognosis in patients with breast (Look et al., 2002), ovarian (Chambers et al., 1998), colon (Sakakibara et al., 2005) and gastric (Sakakibara et al., 2006) cancers. And *SERPINE1* plays multiple roles in human tumorigenesis, such as sustaining proliferative signals (Giacoia et al., 2014), resisting cell death (Schneider et al., 2008), promoting angiogenesis (Bajou et al., 2001), regulating invasion and metastasis (Ma et al., 1997; Brooks et al., 2000), and promoting inflammatory responses (Sawdey and Loskutoff, 1991; Mertens et al., 2006).

Based on the lock and key principle of ligand-receptor interaction, molecular docking can effectively identify small molecular compounds matching the spatial and electrical characteristics of the active site of the target receptor, which provides a basis for further experiments *in vivo* and *in vitro*. These docking results suggested that VD3 may enable *SERPINE1* to target COVID-19. However, the above results were obtained based on bioinformatics and network pharmacology methods. Since this is a virtual screening method, molecular docking involves unpredictable deviations from reality, which may lead to some errors in *in vivo*/*in vitro* experiments. However, the docking results reflect possible therapeutic mechanisms and provide guidance for animal validation experiments.

## Conclusion

In conclusion, the bioinformatics results strongly suggested that antiviral, anti-inflammatory and immunomodulation are key pathways for VD3 in the treatment of STAD/COVID-19. Furthermore, based on the network pharmacology results, we identified effective pharmacological targets of VD3 against STAD/COVID-19, providing new possibilities for further clinical trials.

## Data Availability

The datasets presented in this study can be found in online repositories. The names of the repository/repositories and accession number(s) can be found in the article/Supplementary Material.

## Abbreviations

ACE2, Angiotensin converting enzyme2; BP, biological process; CC, cellular composition; COVID-19, Corona Virus Disease 2019; DEG, differentially expressed genes; GO, Gene Ontology; KEGG, Kyoto Encyclopedia of Genes and Genomes; MF, molecular function; PAI1/SERPINE1, Plasminogen activator inhibitor-1/serpin family E member 1; PPI, Protein-protein interaction; PDB, Protein Data Bank; SARS-CoV-2, Severe acute respiratory syndrome coronavirus 2; STAD, Stomach adenocarcinoma; VD3, vitamin D3.
