# Supplementary material for: Network Pharmacology and Bioinformatics Analyses Identify Intersection Genes of Vitamin D3 and COVID-19 as Potential Therapeutic Targets
Source: Front Pharmacol. 2022 Apr 28;13:874637. doi: 10.3389/fphar.2022.874637 (PMC9095980; doi:10.3389/fphar.2022.874637)
Supplement: Supplementary file 2 [file Table4.pdf]

Supplementary Table 4. DeepSite predition results

PDB ID	Cavity	scores	centers
6LZG	1	0.996699	[-11.4, -0.874, -24.16]
	2	0.932989	[-27.4, 13.13, 5.84]
5R84	1	0.977018	[15.5, 3.65, -4.35]
	2	0.989906	[9.5, -1.65, 23.65]
	3	0.814471	[1.5, -19.65, 9.65]
	4	0.747475	[19.5, 10.35, -0.35]
1A7C	1	0.998783872	[19.63, 21.56, 9.28]
	2	0.995458469	[19.63, 21.56, 19.28]
	3	0.819185138	[19.63, -6.44, 23.28]
