# Supplementary material for: Network Pharmacology and Bioinformatics Analyses Identify Intersection Genes of Vitamin D3 and COVID-19 as Potential Therapeutic Targets
Source: Front Pharmacol. 2022 Apr 28;13:874637. doi: 10.3389/fphar.2022.874637 (PMC9095980; doi:10.3389/fphar.2022.874637)
Supplement: Supplementary file 4 [file Table1.pdf]

Supplementary Table1 Intersecting genes

Symbol	logFC	logCPM	PValue	FDR
ACE2	-3.4348	4.652933	1.32E-35	2.17E-33
APOE	1.774274	8.452704	1.31E-08	9.07E-08
ACE	-2.05242	4.57186	7.61E-38	1.45E-35
CXCL10	2.840116	6.713147	5.05E-11	4.97E-10
CXCL8	3.192488	7.004054	2.28E-12	2.73E-11
ALB	6.551447	6.716306	1.93E-05	0.000078
S100A8	-3.4684	9.036812	3.36E-19	9.9E-18
CALCA	4.112722	3.88141	0.00151	0.004061
S100A9	-3.32313	10.70686	6.88E-21	2.43E-19
GPT	-2.09946	4.052604	4.46E-17	9.85E-16
IL1A	-1.67185	3.19508	2.17E-07	1.24E-06
CXCL1	2.662374	7.354785	8.66E-12	9.47E-11
NCAM1	-2.76619	3.157155	1.88E-26	1.18E-24
PLA2G7	1.99941	4.664355	7.05E-13	9.15E-12
FCGR1A	1.759756	3.214269	1.9E-07	1.09E-06
MAF	-1.6338	4.783415	1.43E-29	1.35E-27
FABP2	-4.5311	4.662591	1.58E-40	3.41E-38
MMP3	4.590926	5.803164	1.28E-12	1.59E-11
CSF2	2.806299	3.059881	1.75E-06	8.45E-06
FCGR3A	2.166347	5.628051	2.32E-13	3.21E-12
SPP1	3.931615	7.819997	1.09E-14	1.8E-13
ELANE	2.075596	3.86379	0.021456	0.042617
IDO1	3.451822	5.804004	2.63E-09	2.02E-08
MPO	2.666926	3.041876	0.00454	0.010829
ANPEP	-2.492	8.737918	8.46E-13	1.08E-11
SERPINE1	2.016549	6.640921	6.8E-10	5.63E-09
CXCR2	-2.0888	3.208276	2.32E-13	3.21E-12
IL1RN	-2.89712	6.886155	5.04E-31	5.43E-29
PTGS1	-1.87733	4.460807	2.57E-29	2.37E-27
RBP4	2.003422	5.812486	0.000158	0.000527
LBP	3.202092	3.319789	1.32E-05	5.47E-05
ADAMTS1	-2.03772	4.969183	5.48E-27	3.7E-25
PRKAR2B	-1.6771	4.037889	1.14E-15	2.13E-14
GDF15	2.623627	6.849782	4.24E-17	9.42E-16
ATP2B4	-1.59527	6.134298	1.77E-15	3.22E-14
MKI67	1.858368	5.78494	1.09E-21	4.21E-20
ZBTB16	-2.68518	3.117729	2.97E-25	1.63E-23
ERBB2	1.747675	7.617832	1.78E-05	7.21E-05
MET	2.116022	6.165509	1.1E-11	1.19E-10
EPHB2	2.006127	4.760394	3.14E-10	2.74E-09
PLAU	2.003859	6.213019	2.53E-14	3.95E-13
MME	-2.45468	3.905106	7.26E-17	1.57E-15

CAV1	-1.53697	6.323286	3.23E-12	3.79E-11
APOB	-4.67319	4.879587	6.56E-29	5.42E-27
FGB	3.211376	4.059977	0.000365	0.001128
RHOB	-1.93557	8.847718	2.04E-30	2.06E-28
HAVCR1	2.870829	3.097959	8.5E-06	3.67E-05
MTHFD1L	1.76698	4.446674	7.18E-22	2.9E-20
NTS	2.779363	5.091462	0.001539	0.004127
KIT	-1.58456	3.813299	1.11E-13	1.6E-12
CKB	-2.59324	7.348584	2.18E-30	2.19E-28
KAT2B	-1.58957	4.292966	1.57E-35	2.52E-33
LTF	-2.66059	6.648502	5.26E-13	6.88E-12
CXCL6	2.51359	4.004898	9.39E-07	4.75E-06
MMP1	1.672913	7.691047	0.000118	0.000404
PRKCG	2.664789	3.307939	4.21E-08	2.67E-07
FGG	2.226889	3.515613	0.025345	0.049431
CHI3L1	2.316964	5.331932	3E-10	2.63E-09
GSTM1	-1.77993	4.379591	4.15E-06	1.88E-05
KLF2	-1.71528	7.253853	2.63E-21	9.86E-20
