# Supplementary material for: Network Pharmacology and Bioinformatics Analyses Identify Intersection Genes of Vitamin D3 and COVID-19 as Potential Therapeutic Targets
Source: Front Pharmacol. 2022 Apr 28;13:874637. doi: 10.3389/fphar.2022.874637 (PMC9095980; doi:10.3389/fphar.2022.874637)
Supplement: Supplementary file 5 [file DataSheet1.pdf]

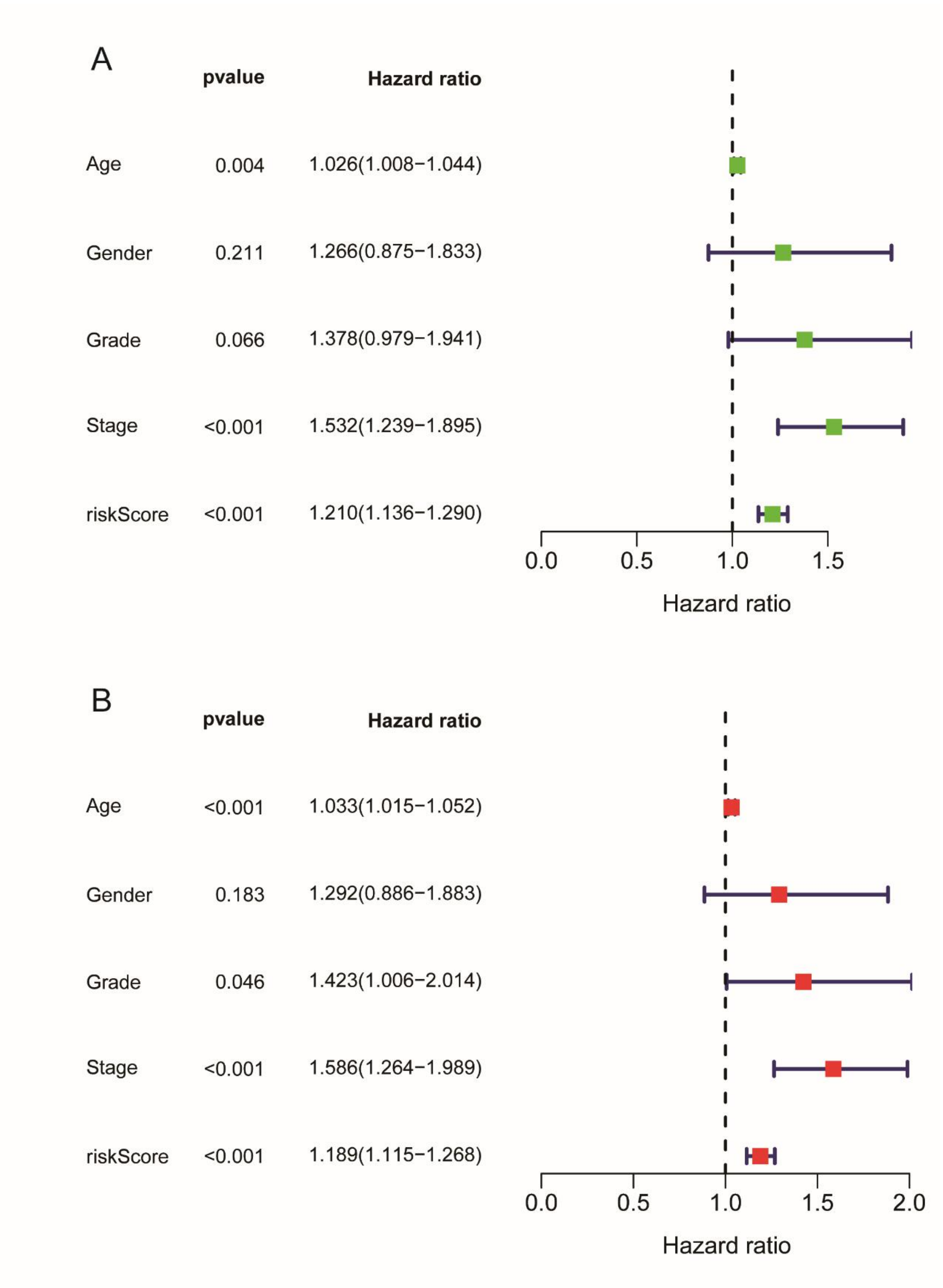

Supplementary figure 1. Univariate cox analysis and multivariate cox analysis for five characteristics

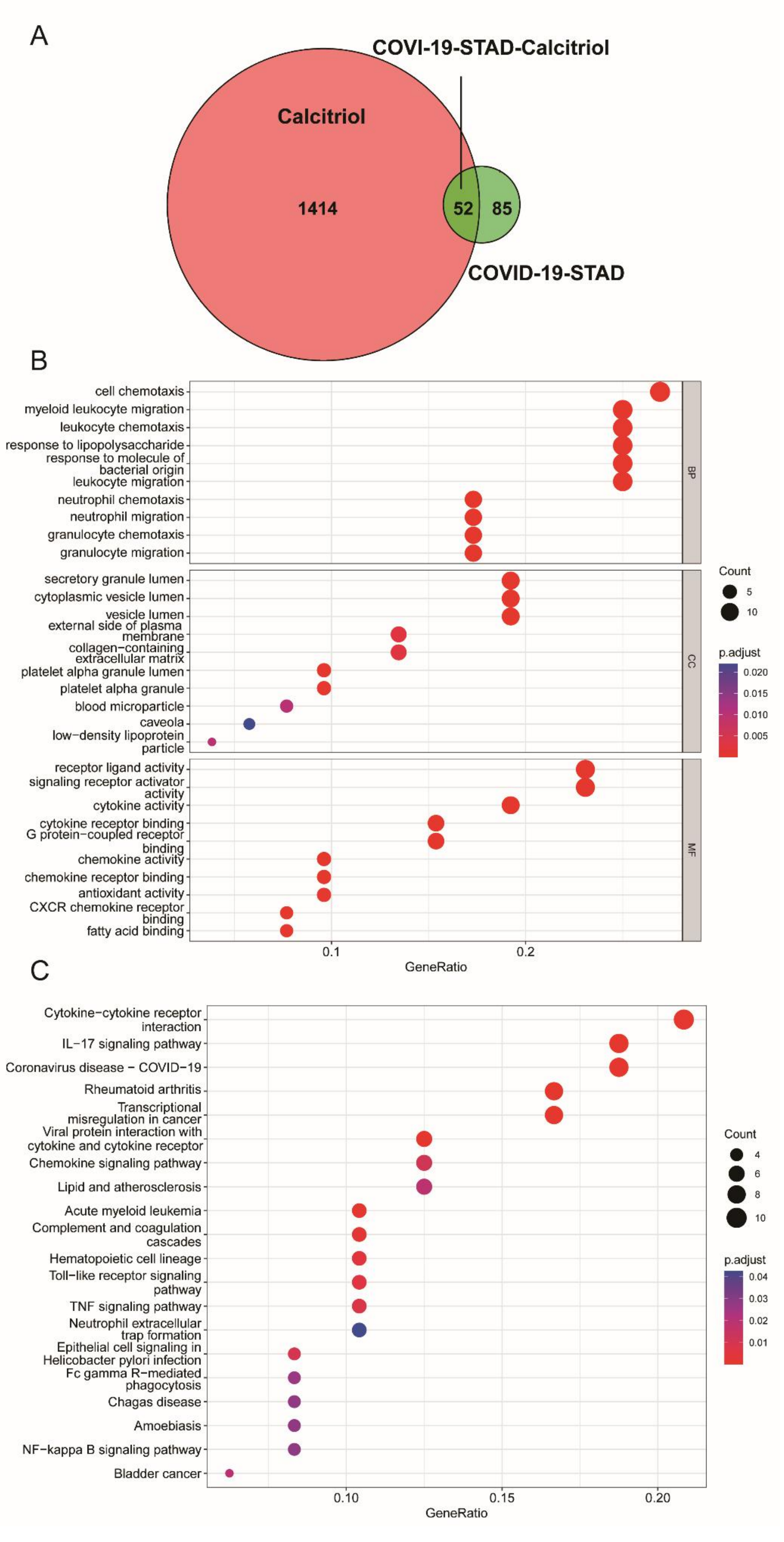

Supplementary figure 2. **(A)** Venn diagram of calcitriol and STAD/COVID-19 Intersecting genes. **(B)** GO analysis of intersecting genes of calcitriol and STAD/COVID-19. **(C)** KEGG pathway enrichment analysis of intersecting genes of calcitriol and STAD/COVID-19.

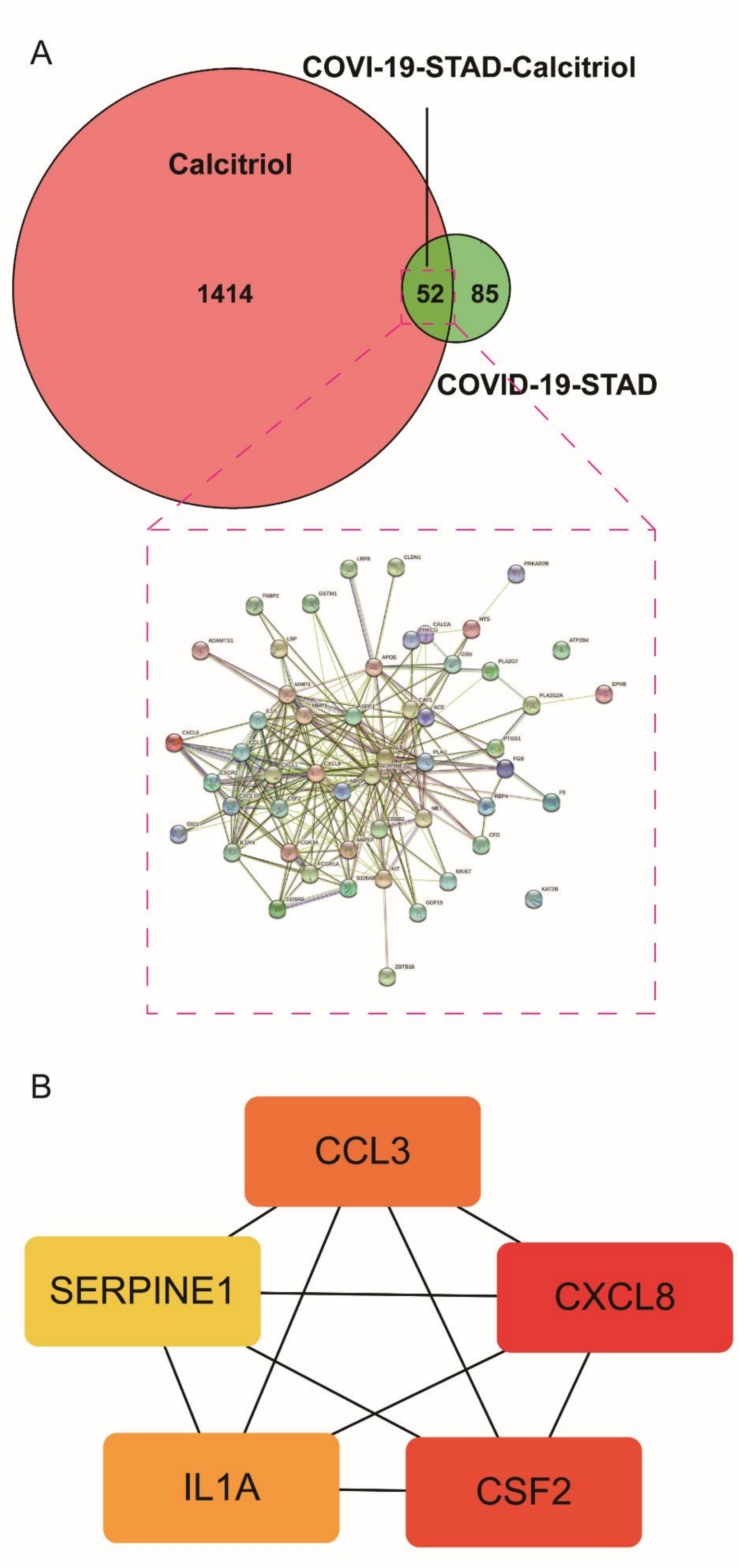

Supplementary figure 3. **(A)** STRING analysis of calcitriol and STAD/COVID-19 intersecting genes. **(B)** Cytoscape analysis of protein interaction networks associated with calcitriol anti-STAD/COVID-19.

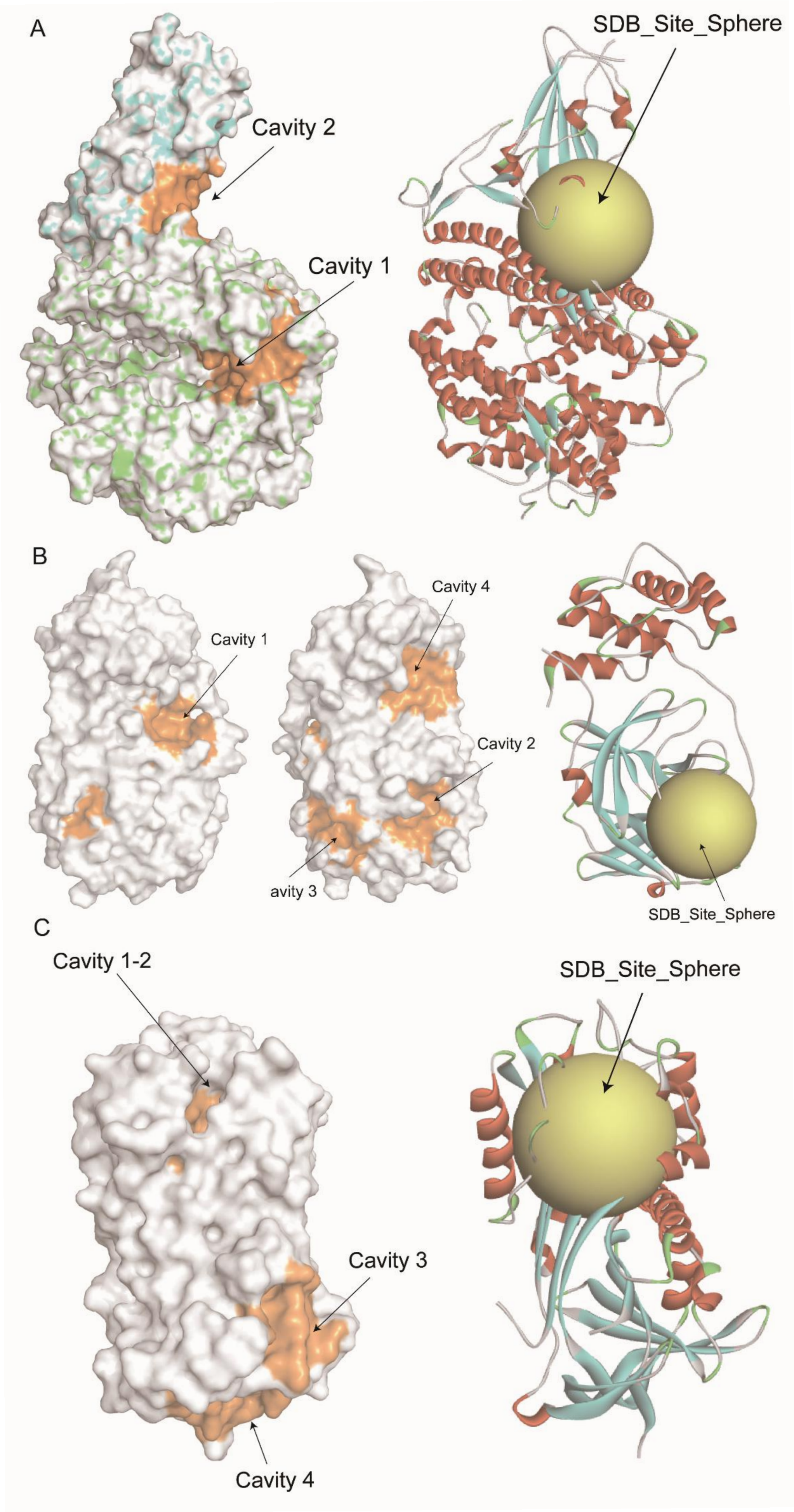
A
SDB_Site_Sphere
Cavity 2
Cavity 1
B
Cavity 1
Cavity 4
Cavity 2
avity 3
SDB_Site_Sphere
C
Cavity 1-2
SDB_Site_Sphere
Cavity 3
Cavity 4

Supplementary figure 4. Identified protein cavity and site information in SARS-COV-2 RBD/ACE2 **(A)**, Mpro **(B)** and PAI1 **(C)**.

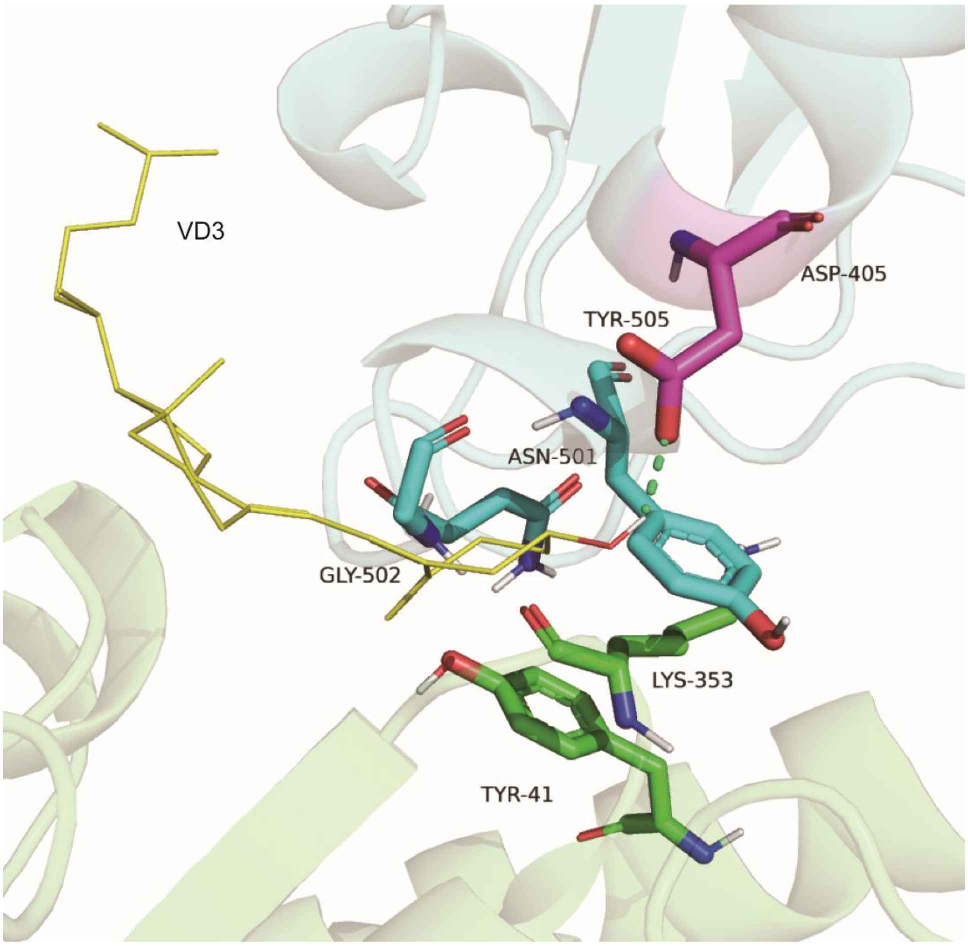

Supplementary figure 5. Partial structure of the SARS-CoV-2 spike receptor-binding domain bound to the ACE2 receptor. ACE2 is shown in green. The SARS-CoV-2 RBD is shown in cyan. VD3 is shown in yellow.

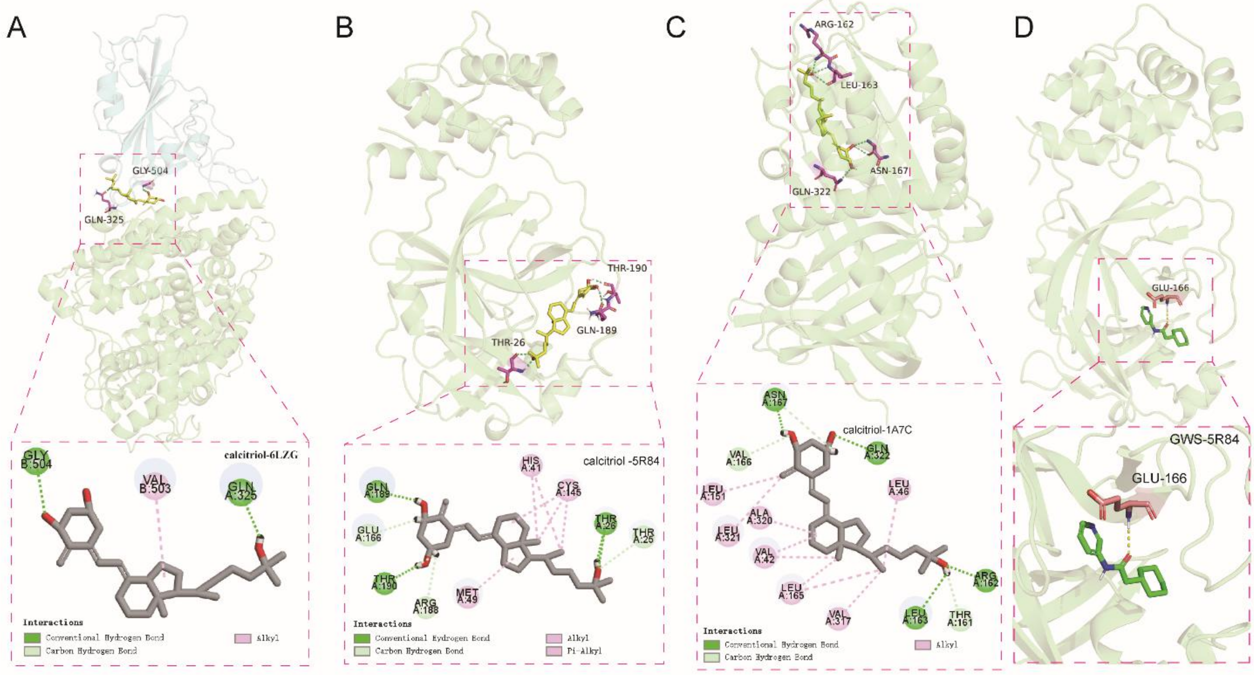

Supplementary figure 6. **(A)** Molecular docking model of calcitriol with SARS-COV-2 Spike RBD/ACE2 complex (PDB: 6LZG, ACE2(green), SARS-CoV-2 RBD (cyan)) and 2D patterns of bonds in the model. **(B)** Molecular docking model of calcitriol with SARS-CoV-2 major protease (PDB: 5R84) and 2D patterns of bonds in the model. **(C)** Molecular docking model of calcitriol with PAI-1 (PDB: 1A7C) and 2D patterns of bonds in the model. **(D)** Molecular docking model of the original non-specific ligands GWS with 5R84 and 3D patterns of bonds in the model.
